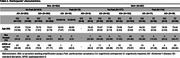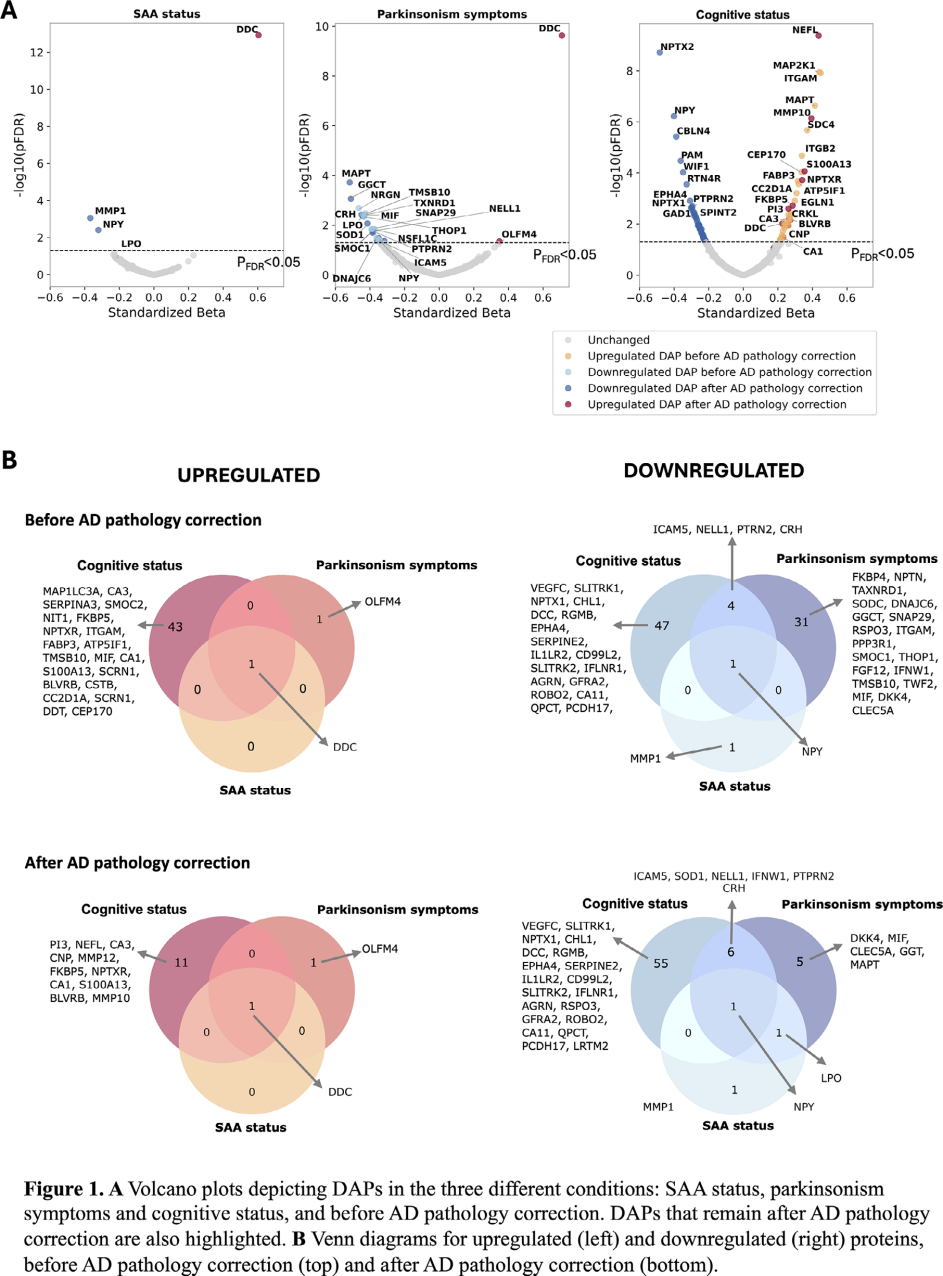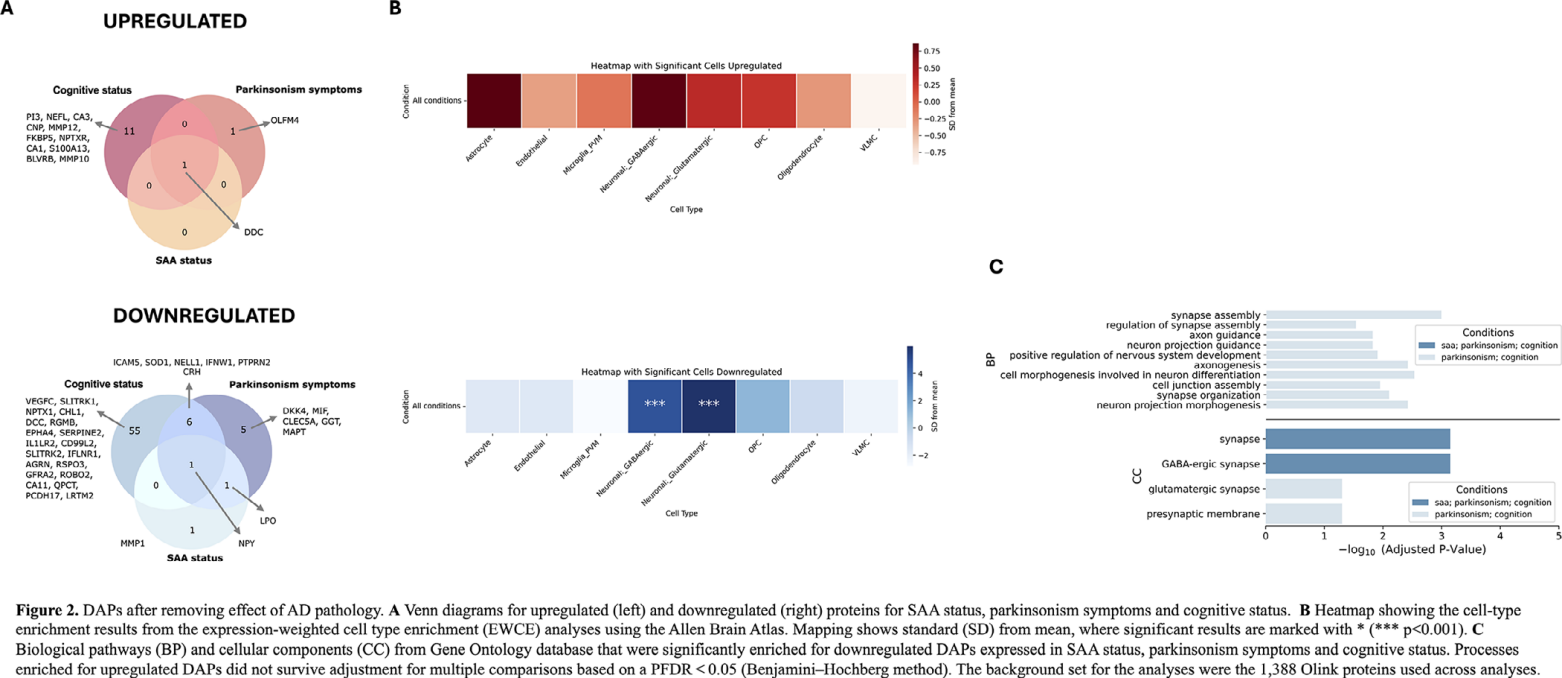# Proteomic profiles in Lewy body pathology and the presence of AD co‐pathology

**DOI:** 10.1002/alz70856_105647

**Published:** 2026-01-08

**Authors:** Irene Cumplido‐Mayoral, Ines Hristovska, Alexa Pichet Binette, Atul Kumar, Olof Strandberg, Shorena Janelidze, Erik Stomrud, Sebastian Palmqvist, Rik Ossenkoppele, Jacob W. Vogel, Niklas Mattsson‐Carlgren, Oskar Hansson

**Affiliations:** ^1^ Clinical Memory Research Unit, Department of Clinical Sciences, Lund University, Lund, Sweden; ^2^ Clinical Memory Research Unit, Department of Clinical Sciences Malmö, Lund University, MOntreal, QC, Canada; ^3^ Centre de Recherche de l’Institut Universitaire de Gériatrie de Montréal, Montréal, QC, Canada; ^4^ Department of Physiology and Pharmacology, Université de Montréal, Montréal, QC, Canada; ^5^ Clinical Memory Research Unit, Department of Clinical Sciences Malmö, Lund University, Lund, Sweden; ^6^ Memory Clinic, Skåne University Hospital, Malmö, Skåne, Sweden; ^7^ Amsterdam Neuroscience, Brain Imaging, Amsterdam, Netherlands; ^8^ Alzheimer Center Amsterdam, Department of Neurology, Amsterdam Neuroscience, Vrije Universiteit Amsterdam, Amsterdam UMC, Amsterdam, Netherlands; ^9^ Department of Clinical Sciences Malmö, SciLifeLab, Lund University, Lund, Sweden; ^10^ Clinical Memory Research Unit, Lund University, Malmö, Skåne, Sweden; ^11^ Wallenberg Center for Molecular Medicine, Lund University, Lund, Sweden

## Abstract

**Background:**

Lewy body diseases (LBD), including Parkinson's disease (PD) and dementia with Lewy bodies (DLB), are neurodegenerative disorders that often overlap with Alzheimer's disease (AD), complicating diagnosis and patient management. The role of α‐synuclein aggregation in LBD and its interplay with AD pathology remains unclear. Biomarkers like DOPA decarboxylase (DDC) have shown promise in differentiating healthy controls, PD, and AD. However, molecular mechanisms underlying disease heterogeneity in LBD require further investigation. Here we examine proteomic differences associated with α‐synuclein pathology and its clinical manifestations, including cognitive decline and parkinsonism symptoms.

**Method:**

We analyzed 1,388 proteins from CSF Olink of 915 participants from the Swedish BioFINDER‐2 cohort, incorporating CSF α‐synuclein seeding amplification assays (SAA) and AD pathology biomarkers (*p*‐tau181/Aβ42). Participants were classified as cognitively unimpaired or impaired (MCI/dementia) and by the presence of parkinsonism. Associations between CSF proteins and SAA pathology, cognitive status, and parkinsonism were assessed using a single linear regression model, adjusting for age, sex, and mean protein levels. Differentially abundant proteins (DAP) across the three conditions (pFDR<0.05) were further evaluated after adjusting for AD pathology. Functional and cell‐type enrichment analyses were performed upregulated and downregulated DAPs separately across conditions. We then identified proteins included in the resulting biological and cellular processes to assess in which conditions they were abundant.

**Result:**

Among 287 SAA+ participants, 110 had parkinsonism symptoms, 198 had cognitive impairment, and 51 were clinically unimpaired (Table 1). We identified 129 DAPs across all conditions: 82 remaining significant after adjusting for AD pathology (Figure 1). Most DAPs were linked to cognitive status, with minimal overlap across conditions (Figure 1B). Downregulated proteins were enriched in synaptic processes, particularly neuronal signaling, cell junction assembly, and axonogenesis (Figure 2). Notably, DDC was consistently upregulated across all conditions, and neuropeptide Y (NPY), involved in stress responses, was consistently downregulated.

**Conclusion:**

We identified specific molecular processes in LBD and their interplay with cognitive and motor symptoms, independently of AD pathology. These suggest disruptions in synaptic and cellular regulation. Furthermore, neuropeptides such as NPY may play a neuroprotective role in LBD. These findings contribute to understanding LBD pathophysiology and identifying potential therapeutic targets.